# Efficacy and Safety of F-Biotic™ in Combination With Metformin for Type 2 Diabetes Mellitus: Results From a Randomized, Double-Blind Clinical Trial

**DOI:** 10.7759/cureus.76053

**Published:** 2024-12-20

**Authors:** Smiju Devassy, Sheejamol S, Santhosh Kumar A K, Balprakash Nair

**Affiliations:** 1 Diabetes and Endocrinology, Arishina Life Sciences, Karnataka, IND; 2 Clinical Studies, Arishina Life Sciences, Karnataka, IND

**Keywords:** diabetes mellitus, glucagon-like peptide 1, glycemic control, prebiotics, quality of life

## Abstract

Introduction: This study evaluated the effectiveness, safety, and tolerability of F-Biotic™ (Arishina Life Sciences, Karnataka, IND), a prebiotic containing 60% resistant starch derived from natural sources, in patients with Type 2 diabetes mellitus (T2DM) on stable metformin therapy.

Methods: Seventy participants with T2DM, aged 25-70 years, were randomized into two groups: one receiving F-Biotic™ and the other a placebo, both administered daily for 12 weeks. Key outcomes included fasting blood sugar (FBS), postprandial blood sugar (PPBS), glycated hemoglobin (HbA1c), fasting insulin, insulin resistance (HOMA-IR), GLP-1 levels, lipid profile, and quality of life.

Results: The F-Biotic™ group exhibited significant reductions in FBS from 150.18 ± 34.78 mg/dL at baseline to 134.24 ± 39.91 mg/dL by the end of the study, compared to an increase in the placebo group from 148.61 ± 42.13 mg/dL to 157.71 ± 47.84 mg/dL. PPBS decreased by −32.97 ± 67.18 mg/dL in the F-Biotic™ group versus an increase of 21.00 ± 36.59 mg/dL in the placebo group. GLP-1 levels increased significantly in the F-Biotic™ group, while they decreased in the placebo group. No significant changes were observed in HbA1c, fasting insulin, or HOMA-IR, and no adverse events related to the investigational product were reported.

Conclusion: F-Biotic™ appears to be a promising adjunctive therapy for T2DM, demonstrating significant improvements in glycemic control and quality of life without adverse events related to the investigational product. Further research with longer follow-ups and larger samples is needed to confirm these findings and evaluate long-term efficacy and safety.

## Introduction

Diabetes mellitus, particularly type 2 diabetes, is a chronic metabolic disorder characterized by persistent hyperglycemia due to impaired insulin secretion, insulin resistance, or a combination of both. The global prevalence of type 2 diabetes has been steadily increasing, posing significant challenges to public health and healthcare systems due to the associated complications, including cardiovascular diseases, neuropathy, nephropathy, and retinopathy [1]. The International Diabetes Federation (IDF) estimates that 537 million people will have diabetes in 2021, with numbers expected to rise to 643 million by 2030 without effective prevention [2].

Current management strategies for type 2 diabetes primarily focus on lifestyle modifications, pharmacotherapy, and, in some cases, surgical interventions. Lifestyle modifications, including dietary changes and increased physical activity, are the cornerstone of diabetes management. These strategies aim to improve glycemic control, reduce insulin resistance, and prevent or delay the onset of complications [3]. Pharmacotherapy includes the use of oral hypoglycemic agents such as metformin, sulfonylureas, and SGLT-2 inhibitors, as well as injectable therapies like insulin and GLP-1 receptor agonists [4]. While these approaches are effective in managing blood glucose levels, they often come with side effects and may not address the underlying causes of insulin resistance and metabolic dysregulation.

Resistant starch (RS), a type of dietary fiber that resists digestion in the small intestine and undergoes fermentation in the large intestine, has emerged as a novel and promising dietary supplement in the management of type 2 diabetes. Unlike conventional strategies that primarily focus on reducing blood glucose levels through pharmacological means or lifestyle changes, resistant starch offers a unique approach by modulating the gut microbiota and improving insulin sensitivity through the production of short-chain fatty acids (SCFAs) like butyrate, acetate, and propionate during fermentation [5].

The fermentation of resistant starch in the colon leads to a range of metabolic benefits, including improved glycemic control, reduced postprandial blood glucose levels, and enhanced insulin sensitivity [6]. These effects are mediated by several mechanisms, such as delayed gastric emptying, reduced glucose absorption in the small intestine, and favorable changes in gut microbiota composition [7]. Unlike pharmacological interventions, which may have adverse effects or lead to hypoglycemia, resistant starch may be a suitable physiological approach to managing diabetes by harnessing the body’s natural metabolic processes [8,9]. Numerous preclinical studies have suggested the use of RS as a sustainable adjuvant in the management of diabetes mellitus [10].

Furthermore, resistant starch differs from other dietary fibers and conventional dietary strategies by its ability to specifically target the large intestine, where its fermentation can induce systemic metabolic effects [11]. This makes it a potentially powerful tool in the dietary management of type 2 diabetes, particularly when integrated with other lifestyle and pharmacological interventions.

This study aims to explore the effectiveness, safety, and tolerability of F-Biotic™ (Arishina Life Sciences, Karnataka, IND), a prebiotic containing 60% resistant starch (RS) derived from natural sources such as fruits (e.g., banana, jackfruit), in subjects with type 2 diabetes mellitus (T2DM) as an adjunct to stable metformin therapy.

## Materials and methods

Study settings and ethical considerations

The study was conducted at a private medical college in Bangalore, India, between February and June 2024. The study was approved by the BGS Global Institute of Medical Sciences Institutional Ethics Committee vide ECR/1307/Inst/KA/2019. All the participants signed an informed consent form before enrollment. The study was registered as a clinical trial in the clinical trial registry of India vide CTRI/2024/03/063627.

Study design

This study was designed as a randomized, double-blind, two-arm, placebo-controlled, parallel-group clinical trial. Participants were randomly assigned to one of two groups: the intervention group receiving the F-Biotic™ and the control group receiving a placebo (Maltodextrin:Corn starch [1:2]).

Study participants

Seventy participants with T2DM who had been on stable metformin monotherapy for at least eight weeks and met all inclusion and exclusion criteria were eligible for the study. Subjects eligible for inclusion in this study were male and female adults aged 25 to 70 years, diagnosed with type 2 diabetes mellitus and on stable metformin monotherapy for at least eight weeks prior to screening. Eligible participants had glycated hemoglobin (HbA1c) levels between 7% and 9%, fasting blood glucose levels of ≥125 mg/dL or postprandial blood sugar ≥140mg/dL, and a body mass index (BMI) between 23-35 kg/m². Females of childbearing potential were required to agree to use approved contraceptive methods during the study and provide a negative urine pregnancy test at screening. All participants were expected to maintain their usual diet, physical activity, and general lifestyle throughout the study period. Additionally, they had to be willing and able to provide informed consent and comply with the study procedures.

Exclusion criteria included a diagnosis of Type 1 diabetes mellitus or the use of medications or supplements other than metformin, known to influence blood glucose levels. Subjects were excluded if they had fasting blood triglyceride levels above 400 mg/dL, low-density lipoprotein (LDL) levels greater than 190 mg/dL, or HbA1c levels above 9% at screening. Individuals with a history of uncontrolled or serious medical conditions, including renal or liver disease, hypertension, diabetic ketoacidosis, cardiac or neurological problems, autoimmune diseases, gastrointestinal bleeding, peptic ulcer disease, psychiatric disorders, or severe neurological conditions, were also excluded. Other exclusion criteria included known allergies to any natural ingredients in the investigational product, immunocompromised status (such as HIV or Hepatitis B positive), pregnancy or lactation, and participation in another clinical trial within the last three months. Finally, any condition that, in the investigator's opinion, could interfere with the subject's treatment, assessment, or compliance with the protocol or that could prevent the subject from completing the study warranted exclusion.

Sample size

A sample size of seventy subjects, thirty-five in each arm, was considered sufficient to detect a clinically important difference between groups with 80% power and a 5% level of significance. This sample size consideration was based on a previous study that examined the efficacy of a 12-week resistant starch supplementation on adults with prediabetes [12].

Randomization

Randomization was performed using block randomization. Each randomized subject received a xx-digit randomization number. Randomized subjects who terminated their study participation for any reason, regardless of whether the IP was taken or not, retained their randomization number. Subjects were randomly assigned to treatment.

Blinding

Blinding was maintained throughout the study, with both the investigator and participants kept unaware of the specific interventions and group allocations.

Study interventions

Participants in the study were divided into two groups, each receiving different interventions. Group A was administered the active intervention, F-Biotic, while Group B received a placebo. Both groups received their respective treatments in sachet form. For Group A, the intervention consisted of an F-Biotic, delivered orally at a dose of 15 g twice a day. Participants were instructed to take the sachets twice a day for a duration of 12 weeks.

Similarly, Group B received placebo sachets containing maltodextrin:corn starch mixed in a ratio of 1:2, matching the dosage and regimen of the F-Biotic group. The placebo was also administered orally at a dose of 5 g per day, taken twice daily for the same 12-week treatment period.

Each participant received a diary card with clear instructions on how and when to take the study medication, as well as a section to record the time and date of each dose. The diary was also used to document any additional medications taken and any changes in health status. Diaries were distributed during the screening or baseline visit, where participants were provided with detailed guidance on how to complete them.

Outcomes

The primary and secondary outcomes of the study were assessed at three key time points: Day 0 (baseline), Day 42, and Day 84 follow-up.

Glycemic Control: Fasting Blood Sugar (FBS), Postprandial Blood Sugar (PPBS), Glycated Hemoglobin (HbA1c), Fasting Insulin, Homeostatic Model Assessment for Insulin Resistance (HOMA-IR), Fasting and Postprandial Glucagon-Like Peptide-1 (GLP-1).

Lipid profile

Anthropometric measurements (height, weight, body mass index, waist circumference, waist-hip ratio).

Safety assessment

Safety assessments included monitoring of adverse events (AEs), physical examination, vital signs measurement, urine pregnancy test (for females of childbearing potential), hematology, and clinical chemistry tests such as complete blood count (CBC), aspartate aminotransferase (AST), alanine aminotransferase (ALT), and serum creatinine. 

In addition to these assessments, the study evaluated participants using the following questionnaires: 1) Gastrointestinal Symptom Rating Scale (GSRS): To assess gastrointestinal symptoms. 2) Quality of Life (QOL): Measured using the Quality of Life in Diabetes (QOLID) questionnaire.

Adverse event monitoring

All noxious and unintended responses to the study product related to any dose were considered ADRs. Subjects were monitored for safety during the study and till the completion of the study by analyzing the AEs. AEs, if any, were evaluated for duration, intensity, and relationship to (or association with) the study treatment (or other causes). Additionally, the actions taken (e.g., discontinuation of study product, administration of treatment) and the resulting outcome of the AE were indicated on the Case Report Form (CRF). All the AEs that occurred during the study were followed and notified to the ethics committee. All AEs were recorded until a subject completed the entire duration of the study.

Participants' withdrawal/termination criteria

Participation was entirely voluntary, allowing any subject to withdraw at any time for any reason by notifying the investigator. If discontinuation was due to medical reasons, subjects were required to remain under medical supervision until their health stabilized or returned to satisfactory levels. The investigator also had the authority to terminate a subject's participation if there was a protocol violation, a medical necessity, or if it was deemed in the best interest of the subject, including in cases of severe or serious adverse events.

Statistical considerations

All the data was analyzed using R software version 4.2.1. Mean differences within and between groups were analyzed at each data collection point using ANOVA for all outcome measures. GSRS and QOLID scores were categorized into domains or scales, with the scores and their changes from baseline summarized by treatment group and visit and assessed using t-tests with a 5% significance level. Continuous outcomes were summarized by treatment and time points using descriptive statistics, including the number of participants (n), mean, median, standard deviation (SD), and minimum, and maximum values. Categorical outcomes were summarized by treatment and time points using frequency counts and percentages. Hypothesis testing was two-sided and conducted at a 0.05 significance level.

**Figure 1:**
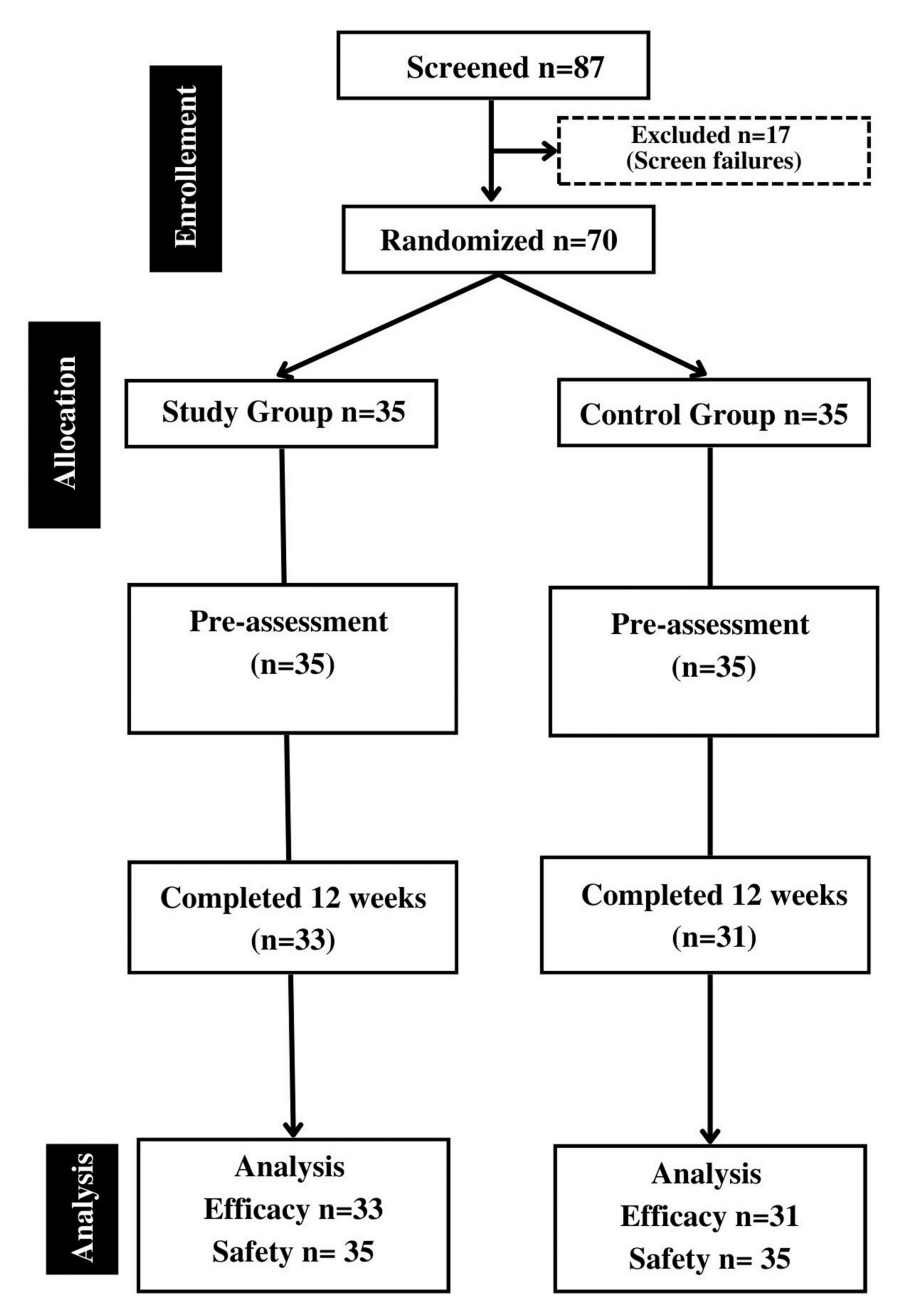
Trial profile

The missing data were not imputed and were excluded from the analyses. All participants recruited into the study were accounted for, including those who did not complete the study, and the reasons for their withdrawal were documented. Participants were included in the intent-to-treat population if they had taken the investigational product for at least one day and had completed at least one post-baseline assessment. The CONSORT trial profile is depicted in Figure 1.

## Results

Out of 87 subjects screened for the study, 17 were identified as screen failures. A total of 70 subjects were randomized, with 64 participants completing the study and being included in the efficacy analysis. The demographic characteristics of the participants are tabulated in Table 1. The detailed results are discussed below.

**Table 1:** Demographic and baseline characteristics of study participants

Variables	Interventional group (n=33) (Mean± SD)	Placebo group (n=31) (Mean± SD)
Age	47.18 (10.77)	50.06 (9.86)
Height (cm)	159.4 (9.12)	159.45 (9.87)
Weight (kg)	69.23 (10.26)	69.91 (7.84)
BMI (kg/m^2^)	27.24 (3.29)	27.60 (3.19)
Waist Circumference (cm)	99.94 (6.52)	99.29 (6.75)
Hip Circumference (cm)	99.85 (6.04)	100.77 (6.25)
Gender	Male 19 (30%)	Male 14 (22%)
	Female 14 (22%)	Female 17 (27%)

Changes in fasting blood sugar (FBS)

The interventional group showed a significant reduction in FBS levels, decreasing from a baseline of 150.18 ± 34.78 mg/dL to 140.49 ± 29.04 mg/dL at day 42 and further to 134.24 ± 39.91 mg/dL by the end of the study (day 84). However, in the placebo group, the subjects showed an increase in the FBS from baseline (148.61 ± 42.13 mg/dL) to day 42 (154.29 ± 38.95 mg/dL) to the end of the study (157.71 ± 47.84 mg/dL). The change in the FBS from baseline to day 42 was not statistically significant in the F-Biotic group ( −9.70 ± 24.48 mg/dL) compared to the placebo group (5.68 ± 23.93 mg/dL). The change in the FBS from baseline to the end of the study was significant in the F-Biotic group (p<0.05, −15.94 ± 29.34 mg/dL) compared to the placebo group (9.10 ± 29.34 mg/dL). The results are shown in Table 2.

**Table 2:** Summary of the changes in outcome measures across time points *Significant  p<0.05; SD: Standard deviation

Variables	Time points	Interventional group (Mean ± SD)	Confidence Interval 95%	Placebo group (Mean ± SD)	Confidence Interval 95%	P value	Effect Size
FBS (mg/dL)	Baseline	150.18±34.78	(82.01;218.34)	148.61±42.13	(66.03; 231.18)	0.871	0.041
	Day 42	140.49± 29.04^*^	(83.57;197.40)	154.29±38.95	(77.94; 230.63)	0.112	-0.402
	Day 84	134.24±39.91^*^	(56.01;212.46)	157.71±47.84	(63.94; 251.47)	0.037*	-0.533
PPBS (mg/dL)	Baseline	210.00 ± 50.30	(111.41; 308.58)	217.39 ± 50.91	(117.60; 317.17)	0.562	-0.146
	Day 42	192.24 ± 63.19^*^	(68.38; 316.09)	225.10 ± 39.19	(148.28; 301.91)	0.016*	-0.625 -1.133
	Day 84	177.03 ± 66.03^*^	(47.61; 306.44)	238.39 ± 38.76	(162.42; 314.35)	0.000*	
HbA1C (%)	Baseline	8.28 ± 0.62	(7.06; 9.49)	8.18 ± 0.63	(6.94; 9.41)	0.505	0.160
	Day 42	7.83 ± 1.24^*^	(5.39; 10.26)	8.39 ± 0.81	(6.80; 9.97)	0.039*	-0.535
	Day 84	7.55 ± 1.37^*^	(4.86; 10.23)	8.66 ± 1.20	(6.30; 11.01)	0.001*	-0.862
Fasting insulin (uIU/mL)	Baseline	21.53 ± 12.51	(-2.98; 46.04)	20.79 ± 8.19	(4.73; 36.84)	0.780	0.070
	Day 42	21.67 ± 11.61	(-1.08; 44.42)	20.77 ± 8.06	(4.97; 36.56)	0.722	0.090
	Day 84	21.15 ± 12.06	(-2.48; 44.78)	20.43 ± 8.10	(4.55; 36.30)	0.783	0.070
Insulin resistance (HOMA-IR)	Baseline	7.99 ± 4.93	(-1.67; 17.65)	8.28 ± 5.50	(-2.5; 19.06)	0.826	-0.056
	Day 42	7.58 ± 4.26	(-0.76; 15.92)	8.50 ± 5.15	(-1.59; 18.59)	0.862	-0.195
	Day 84	7.41 ± 5.70	(-3.76; 18.58)	8.68 ± 6.21	(-3.57; 20.93)	0.655	-0.212
GLP-1	Baseline	38.39 ± 12.75	(13.4; 63.38)	38.82 ± 10.22	(18.78; 58.85)	0.885	-0.037
	Day 42	39.12 ± 12.87^*^	(13.89; 64.34)	38.61 ± 10.24*	(18.53; 58.68)	0.862	0.044
	Day 84	39.76 ± 13.11^*^	(14.06; 65.45)	38.43 ± 10.25*	(18.34; 58.52)	0.655	0.113
Total cholesterol (mg/dL)	Baseline	180.55±35.36	(111.24; 249.85)	178.65±37.28	(105.581 251.71)	0.575	0.052
	Day 42	175.63±32.38	(112.16; 239.09)	180.42±33.65	(114.46; 246.37)	0.890	-0.145
	Day 84	172.76±32.33	(109.39; 236.12)	185.90±36.08	(115.18; 256.61)	0.540	-0.384
Triglycerides (mg/dL)	Baseline	170.09±57.01	(58.35; 281.82)	169.71±57.49	(57.02; 282.39)	0.001*	0.007
	Day 42	168.36±52.80	(64.87; 271.84)	181.55±68.59	(47.11; 315.98)	0.735	-0.216
	Day 84	166.45±55.63	(57.41; 275.48)	188.90±62.61*	(66.18; 311.61)	0.731	-0.379
HDL (mg/dL)	Baseline	41.85±9.17	(23.87; 59.82)	38.77±7.52	(24.03; 53.50)	0.483	0.367
	Day 42	41.85±7.91	(26.34; 57.35)	44.03±8.83*	(26.72; 61.33)	0.017*	-0.260
	Day 84	43.91±10.54	(23.25; 64.56)	41.39±7.33	(27.02; 55.75)	0.415	0.278
LDL (mg/dL)	Baseline	104.67±34.19	(37.65; 171.68)	105.97±35.19	(36.99; 174.94)	0.465	-0.037
	Day 42	100.00±30.70	(39.82; 160.17)	100.06±33.09	(35.20; 164.91)	0.369	-0.002
	Day 84	95.52±26.73	(43.12; 147.91)	106.74±34.01	(40.08; 173.39)	0.583	-0.367
VLDL (mg/dL)	Baseline	34.03±11.38	(11.72; 56.33)	33.90±11.53	(11.30; 56.49)	0.903	0.011
	Day 42	33.79±10.59	(13.03; 54.54)	36.32±13.79	(9.29; 63.34)	0.621	-0.206
	Day 84	33.33±11.06	(11.65; 55.00)	37.77±12.62	(13.03; 62.50)	0.599	-0.374

Changes in postprandial blood sugar (PPBS)

The intervention group showed a significant reduction in the PPBS across all time points. However, there was an increase in the PPBS among the placebo group across time points. The change in the postprandial blood sugar from baseline to the end of the study was significant in the intervention group (p < 0.05, −32.97 ± 67.18 mg/dL) compared to the placebo group (21.00 ± 36.59 mg/dL). The results are tabulated in Table 2.

Changes in glycosylated hemoglobin (HbA1C)

Similar to the findings for PPBS, the intervention group demonstrated a significant reduction in HbA1c levels across all time points, whereas the placebo group showed an increase in HbA1c levels. The intervention group has shown statistically significant changes in the second and third follow-ups. The results are tabulated in Table 2.

Changes in fasting insulin

Both intervention groups did not exhibit significant changes when compared either within the groups or between them. The results are presented in Table 2.

Changes in insulin resistance (HOMA-IR)

Participants in the intervention group exhibited a decrease in HOMA-IR levels from baseline to the end of the study, while the placebo group showed an increase over the same period. However, the differences between the groups were not statistically significant. The results are presented in Table 2.

Changes in insulin sensitivity by assessing GLP-1 levels

Participants in the intervention group demonstrated a significant increase in GLP-1 levels from baseline to the end of the study, reflecting a positive effect of the treatment. Conversely, the placebo group experienced a significant decrease in GLP-1 levels over the same period, indicating no beneficial impact. The changes in GLP-1 levels from baseline to both Visit 2 and the study's conclusion were significantly more favorable in the intervention group compared to the placebo group (Table 2).

Changes in lipid profile

Participants in the intervention group did not show any significant changes in lipid profile from baseline to the end of the study. However, those in the placebo group exhibited significant changes in parameters such as triglycerides, HDL, VLDL, and the CHOL/HDL ratio. Nevertheless, the group analysis has shown no statistically significant changes (Table 2).

Changes in gastrointestinal health

Participants in both the intervention and placebo groups showed no significant change in the overall GSRS score from baseline to the end of the study, with no significant differences observed between the groups. However, in the intervention group, there was a trend towards a reduction in abdominal pain and constipation scores (Table 3).

**Table 3:** Summary of changes in gastrointestinal health and quality of life among the study participants *Significant  p<0.05; SD: Standard deviation

Variables	Time points	Interventional group (Mean ± SD)	Confidence Interval 95%	Placebo group (Mean ± SD)	Confidence Interval 95%	p-value	Effect Size
Gastrointestinal health score							
Reflux score	Baseline	1.55±1.01	(-0.42; 3.52)	1.65±0.91	(-0.1336; 3.4336)	0.680	-0.104
	Day 42	1.59± 0.96	(-0.29; 3.47)	1.65±0.62	(0.4648; 2.8952)	0.790	-0.111
	Day 84	1.44±0.60	(0.26; 2.61)	1.60±0.60	(0.424; 2.776)	0.296	-0.267
Abdominal Pain Score	Baseline	1.40±0.50	(0.42; 2.38)	1.38±0.48	(0.4392; 2.3208)	0.822	0.041
	Day 42	1.33±0.47	(0.40; 2.25)	1.19±0.21	(0.7784; 1.6016)	0.132	0.385
	Day 84	1.22±0.27	(0.69; 1.74)	1.17±0.19	(0.7976; 1.5424)	0.399	0.214
Indigestion Score	Baseline	1.45±0.73	(0.019; 2.88)	1.51±0.76	(0.02; 2.99)	0.743	-0.081
	Day 42	1.43±0.66	(0.13; 2.72)	1.65±1.04	(-0.38; 3.68)	0.328	-0.253
	Day 84	1.42±0.64	(0.16; 2.67)	1.59±0.90	(-0.17; 3.35)	0.399	-0.218
Diarrhea Score	Baseline	1.11±0.23	(0.65; 1.56)	1.12±0.25	(0.63; 1.61)	0.905	-0.042
	Day 42	1.15±0.28	(0.60; 1.69)	1.03±0.10	(0.83; 1.22)	0.028*	0.571
	Day 84	1.15±0.28	(0.60; 1.69)	1.03±0.10*	(0.83; 1.22)	0.028*	0.571
Constipation Score	Baseline	1.53±0.92	(-0.27; 3.33)	1.44±0.57	(0.32; 2.55)	0.642	0.118
	Day 42	1.45±0.80	(-0.11; 3.01)	1.62±0.68	(0.28; 2.95)	0.340	-0.229
	Day 84	1.35±0.66	(0.05; 2.64)	1.60±0.62	(0.38; 2.81)	0.124	-0.390
GSRS Score	Baseline	1.41±0.56	(0.31; 2.50)	1.42±0.40	(0.63; 2.20)	0.929	-0.021
	Day 42	1.39±0.51	(0.39; 2.38)	1.43±0.34	(0.76; 2.09)	0.729	-0.092
	Day 84	1.32±0.37	(0.59; 2.04)	1.40±0.30	(0.81; 1.98)	0.342	-0.238
Quality of life scores							
Overall Score - QOLID	Baseline	74.6±7.15	(60.58; 88.61)	75.32±6.75	(62.09; 88.55)	0.101	-0.104
	Day 42	77.48±7.5*	(62.78; 92.18)	74.78±6.77	(61.51; 88.04)	0.780	0.378
	Day 84	79.58±5.74	(68.32; 90.83)	74.63±6.98	(60.94; 88.31)	0.000*	0.775

Changes in quality of life

Participants in the intervention group showed a significant improvement in quality-of-life scores for Indian diabetes patients, with scores increasing from baseline to Visit 2 and further to the end of the study. In contrast, participants in the placebo group experienced a decrease in these quality-of-life scores over the same period. The changes in quality-of-life scores from baseline to both Visit 2 and the end of the study were significantly greater in the intervention group compared to the placebo group (Table 3).

Adverse events

During the study period, a total of 13 adverse events (AEs) were reported, with five occurring in the intervention group and eight in the placebo group. All reported AEs were mild in severity and were deemed by the investigator to be unrelated to the investigational product. The AEs included fever, headache, tiredness, cold, gastritis, and body aches, with fever being the most frequently reported (Table 4). All AEs resolved before the end of the study. No participants experienced serious adverse events (SAEs) or were withdrawn from the study due to AEs or SAEs. The safety data suggests that resistant starch is safe to use and well tolerated by the participants.

**Table 4:** Adverse events observed among study participants

Adverse Events Term	GROUP A (F-Biotic, n = 35) n (%)	GROUP B (Placebo, n = 35) n (%)	TOTAL (N =70) n (%)
Subjects with at least one AE	3 (8.57 %)	5 (14.28 %)	8 (11.42 %)
Total number of AE reported	05	08	13
Fever	01 (2.85 %)	03 (8.57 %)	04 (5.71 %)
Headache	02 (5.71 %)	00	02 (2.85 %)
Tiredness	01 (2.85 %)	01 (2.85 %)	02 (2.85 %)
Cold	01 (2.85 %)	02 (5.71 %)	03 (4.28 %)
Gastritis	00	01 (2.85 %)	01 (1.42 %)
Body ache	00	01 (2.85 %)	01 (1.42 %)

## Discussion

This study aims to explore the effectiveness, safety, and tolerability of F-Biotic™, a prebiotic formulation containing 60% resistant starch (RS) derived from natural sources such as bananas and jackfruit, in subjects with type 2 diabetes mellitus (T2DM) as an adjunct to stable metformin therapy. Resistant starch is known for its the potential to improve glycemic control by modulating gut microbiota and enhancing insulin sensitivity, making it a promising candidate for diabetes management.

The intervention group, receiving F-Biotic™, showed a significant reduction in fasting blood sugar (FBS) and postprandial blood sugar (PPBS) levels, which supports its effectiveness in improving glycemic control. This is in line with the known effects of resistant starch on glucose metabolism, as RS can promote increased production of short-chain fatty acids and improve gut health, leading to better blood glucose management. Previous studies have indicated that prebiotics like resistant starch can beneficially influence blood glucose levels by enhancing insulin sensitivity and reducing glycemic fluctuations [13,14].

Despite these improvements in FBS and PPBS, changes in glycosylated hemoglobin (HbA1c) levels were not statistically significant. This finding suggests that while F-Biotic™ may offer short-term benefits in glycemic control, its long-term impact on HbA1c might require further investigation. The variability in HbA1c response could be attributed to the study duration or the fact that HbA1c is influenced by a range of factors over a longer period [14].

Additionally, while no significant changes were observed in fasting insulin levels or insulin resistance (HOMA-IR), the increase in GLP-1 levels in the intervention group suggests that F-Biotic™ may positively influence insulin sensitivity through this pathway. GLP-1 is an important hormone that enhances insulin secretion in response to meals, and its increase could reflect an improvement in glucose metabolism [15].

In terms of safety and tolerability, the study reported mild adverse events, with a higher incidence in the placebo group, and no serious adverse events. This indicates that F-Biotic™ is generally well-tolerated, which aligns with safety profiles reported for similar prebiotic interventions [16]. The absence of significant gastrointestinal disturbances or other adverse effects further supports the safety of using F-Biotic™ in conjunction with metformin therapy.

Quality-of-life improvements in the intervention group, contrasted with declines in the placebo group, highlight the broader benefits of F-Biotic™ beyond glycemic control. Enhanced quality of life is an important aspect of chronic disease management and underscores the potential value of F-Biotic™ as an adjunctive therapy for diabetes management. The results are in agreement with the previous studies that discuss the efficacy of probiotics in the management of type 2 diabetes mellitus [16,17].

Overall, the study provides evidence that F-Biotic™, with its high content of resistant starch from natural sources, is an effective and well-tolerated adjunct to stable metformin therapy in patients with type 2 diabetes mellitus. The positive outcomes observed in glycemic control and quality of life suggest that F-Biotic™ has potential as a beneficial addition to diabetes care regimens. Further research with longer follow-up periods and larger sample sizes will be valuable in confirming these findings and elucidating the underlying mechanisms of action.

The study's strengths include its rigorous inclusion and exclusion criteria, which ensured that participants were well-defined and representative of individuals with type 2 diabetes mellitus (T2DM) on stable metformin therapy. This methodological rigor enhances the reliability of the results by minimizing potential confounding variables. Additionally, the randomized, double-blind, placebo-controlled design is a robust approach that reduces biases, allowing for a clear comparison between the F-Biotic™ intervention and placebo. The comprehensive assessment of various outcomes, such as glycemic control, lipid profile, anthropometric measurements, and quality of life, provides a thorough evaluation of the intervention’s effects. The detailed monitoring of adverse events further contributes to a comprehensive safety profile of F-Biotic™, enhancing the credibility of the safety assessment.

However, the study has some limitations. The 12-week duration may be insufficient to capture long-term effects on HbA1c levels and other chronic diabetes-related outcomes, suggesting a need for longer studies to evaluate sustained efficacy and safety. The sample size of 70 participants may limit the study’s power to detect small but clinically significant differences, and the homogeneous participant demographic could affect the generalizability of the results. Additionally, the study does not address long-term impacts on diabetes management or complications, and despite blinding, participant behavior or reporting could still be influenced by awareness of treatment assignment. These limitations should be considered when interpreting the results and in the design of future research to further explore the efficacy and safety of F-Biotic™.

## Conclusions

In conclusion, F-Biotic™ has shown significant promise in enhancing blood sugar levels and improving the overall quality of life for patients with Type 2 diabetes who are also undergoing metformin treatment. The observed benefits suggest that F-Biotic™ could play a valuable role in managing this chronic condition, potentially offering a complementary approach alongside traditional medications. While the results from this study are encouraging, they underscore the need for further research to validate these findings. Larger sample sizes and longer follow-up periods are essential to assess the long-term efficacy and safety of F-Biotic™. Ultimately, establishing the full benefits and limitations of F-Biotic™ will be crucial for healthcare providers in order to make informed recommendations for their patients. By expanding our understanding through rigorous studies, we can better support individuals living with Type 2 diabetes and enhance their treatment options for improved health outcomes.
